# Health-related quality of life in living kidney donors participating in kidney exchange programmes

**DOI:** 10.1093/ckj/sfae374

**Published:** 2024-11-23

**Authors:** Stijn C van de Laar, Berwout W Wiltschut, Chris A J Oudmaijer, Kelly Muller, Emma K Massey, Robert J Porte, Frank J M F Dor, Robert C Minnee

**Affiliations:** Erasmus MC Transplant Institute, Department of Surgery, Division of HPB & Transplant Surgery, Erasmus University Medical Center, Rotterdam, The Netherlands; Imperial College Renal and Transplant Centre, Hammersmith Hospital, Imperial College Healthcare NHS Trust, London, UK; Erasmus MC Transplant Institute, Department of Surgery, Division of HPB & Transplant Surgery, Erasmus University Medical Center, Rotterdam, The Netherlands; Erasmus MC Transplant Institute, Department of Surgery, Division of HPB & Transplant Surgery, Erasmus University Medical Center, Rotterdam, The Netherlands; Erasmus MC Transplant Institute, Department of Surgery, Division of HPB & Transplant Surgery, Erasmus University Medical Center, Rotterdam, The Netherlands; Erasmus MC Transplant Institute, Department of Internal Medicine, Nephrology and Transplantation, Erasmus Medical Center, Rotterdam, The Netherlands; Erasmus MC Transplant Institute, Department of Surgery, Division of HPB & Transplant Surgery, Erasmus University Medical Center, Rotterdam, The Netherlands; Imperial College Renal and Transplant Centre, Hammersmith Hospital, Imperial College Healthcare NHS Trust, London, UK; Department of Surgery and Cancer, Imperial College, London, UK; Erasmus MC Transplant Institute, Department of Surgery, Division of HPB & Transplant Surgery, Erasmus University Medical Center, Rotterdam, The Netherlands

**Keywords:** health-related quality of life, kidney exchange programme, living donor kidney transplantation

## Abstract

**Background:**

Kidney exchange programmes (KEPs) have revolutionized living donor kidney transplantation (LDKT) by enabling transplants for patients with HLA- or ABO-incompatible donors. However, the implications for donors participating in KEPs, particularly regarding postoperative health-related quality of life (HRQoL), are not well elucidated. This study compares the HRQoL of donors participating in KEPs with donors donating directly (non-KEPs).

**Methods:**

The study included 724 donors, with 121 in the KEP group and 603 in the non-KEP group. Outcomes were assessed using the mental component summary (MCS), physical component summary (PCS), EQ-5D-3L, MVI-20 score, and self-rated pain level. We used a mixed-effects regression model to assess differences between KEP and non-KEP over time, accounting for repeated measures within subjects.

**Results:**

There was a significant temporary decline in both the MCS and PCS post-donation; however, these outcomes returned to pre-donation levels after an interval of 2 months. Donors participating in the KEP had higher PCS and MCS 1-year post-donation. Comparable results were observed in the self-assessed HRQoL using the EQ-5D-3L instrument, as well as in the fatigue scores measured by the MVI-20.

**Conclusions:**

We found that participation in KEPs does not adversely affect donors' short- or long-term mental and physical HRQoL outcomes and that LDKT donors have HRQoL of pre-donation levels soon after donation. These insights are reassuring, indicating that donors participating in KEPs can expect HRQoL comparable to those who do not. This reinforces the viability of KEPs as a safe option for expanding LDKT and findings can inform patient and donor education.

KEY LEARNING POINTS **What was known:**

- Living kidney donors typically maintain good health-related quality of life post-donation, though fatigue and temporary declines in physical and mental health are common. Previous studies have not differentiated HRQoL outcomes between donors participating in kidney exchange programmes (KEPs) and those donating directly to intended recipients.

**This study adds:**

- This study demonstrates that living kidney donors in KEPs experience comparable or better HRQoL outcomes compared with non-KEP donors. Fatigue levels return to pre-donation status within 2 weeks. These findings suggest no significant adverse effects on HRQoL from participating in KEPs.

**Potential impact:**

- Reassuring potential donors about HRQoL outcomes may encourage more individuals to participate in KEPs, thereby increasing living donor kidney transplantation rates. The study supports implementing KEPs in living donor kidney transplantation programmes globally and suggests that either transporting the kidney or having the donor travel are viable options.

## INTRODUCTION

End-stage kidney disease is a debilitating condition affecting millions of people worldwide with a global prevalence of 9.1% [1, 2]. Living donor kidney transplantation (LDKT) is considered the most effective treatment for this condition [3, 4], resulting in better graft survival and economic benefits compared with deceased donor kidney transplantation [5] or dialysis [6, 7]. Notably pre-emptive transplantation is associated with the most preferable outcomes [8, 9]. However, transplant opportunities between donor–recipient pairs are often hampered by HLA or blood group incompatibility. Kidney exchange programmes (KEPs) have emerged as a solution to this challenge [10] with excellent outcomes [11]. KEPs offer the benefits of LDKT for incompatible donor–recipient pairs or for compatible pairs seeking a better HLA or age match. KEP chains are initiated by either unspecified, also referred to as anonymous or non-directed, donors [12, 13] or another donor–recipient pair. This approach has led to a significant increase in the number of successful living donor kidney transplants (LDKTs), particularly for individuals who could have otherwise waited years for a transplant [11].

Studies have shown that KEPs provide outcomes comparable to those of non-KEP LDKT in terms of graft survival and showed that transportation of the kidney graft does not hamper transplant outcomes [11]. Although postoperative complications occur only in 7.3% of donors and the reported mortality rate was 0.01% [14], we do not know whether donors experience a decline in health-related quality of life (HRQoL) following live donor nephrectomy [15]. Studies showed that living kidney donors generally experience a return to pre-donation HRQoL following donation [16]. However, the unique circumstances of KEPs suggest that more research is needed to fully document the impact of this approach on donors. These circumstances include the fact that the donor is donating to an anonymous recipient and may experience anxiety or stress related to travel or complications with the transplant or matching runs. Additionally, the fact that these patients are harder to match because of higher sensitization increases the waiting time before a match is found. The nuance is important since donors do not donate directly to their intended recipient and in some countries, such as the Netherlands, Canada, and Slovakia, donors must travel to their matched recipient's hospital, where an unfamiliar surgical team performs the donor nephrectomy. Furthermore, travel might be logistically and financially challenging for some donors, the donor and intended recipient are separated during a time when they need each other's support, and donor follow-up becomes more difficult [17]. Finally, the support network around the pair is needed in two places at once.

In this study, we aimed to address the hiatus in the literature regarding donor HRQoL in KEP by studying the following research questions in our retrospective study: (i) whether the donor's HRQoL decreases after kidney donation compared with pre-nephrectomy HRQoL, and (ii) whether the donor's HRQoL is comparable in KEP versus non-KEP after living donor nephrectomy.

## MATERIALS AND METHODS

### Study design

The study was a non-randomized, prospective cohort study that included all adult living kidney donors who donated kidneys between 2014 and 2022 at the Erasmus MC Transplant Institute in Rotterdam, the Netherlands. We conducted and reported this study in accordance with the STROBE guidelines [18].

### Setting

The Erasmus MC Transplant Institute, located in Rotterdam, the Netherlands, served as the setting for the study, where multiple surgical techniques for kidney donation have been employed over time, including mini-open, laparoscopic, and robot-assisted nephrectomy.

### Participants

All adult living kidney donors that donated between 2014 and 2022 at the Erasmus MC Transplant Institute in Rotterdam, the Netherlands, were included in the study. All donors were preoperatively worked up by a nephrologist, nurse practitioner, transplant surgeon, medical psychologist on indication, anaesthetist, and a cardiologist on indication. Before being enrolled in the study, all donors were fully informed about the research and its objectives. They provided their explicit written informed consent, agreeing to participate and to the use of their contact information for the distribution of study-related questionnaires. This consent was obtained in accordance with ethical guidelines to ensure the protection of the donors' personal information and their rights as study participants. Participants were excluded from the study either if they did not complete the initial pre-donation questionnaire, if they did not provide the requisite follow-up questionnaire data, if they did not agree to provide informed consent, or if (non-donation related) disabilities prevented them from participating. Questionnaires were filled in on paper and sent by post at regular time points up to 1 year post-nephrectomy.

Over time, various surgical techniques have been employed for donor nephrectomy. Initially, transperitoneal laparoscopic donor nephrectomy was introduced in 1997. This was followed by mini-open donor nephrectomy, which was commonly performed from 2001 to 2006. Starting in 2007, hand-assisted laparoscopic donor nephrectomy became a standard practice. Subsequently, robot-assisted nephrectomy was adopted [19–24]. In the last 5 years the laparoscopic procedure has become the preferred technique. Postoperatively, donors received patient-controlled analgesia enabling the donor to administer intravenous morphine by pressing a button. Furthermore, 1000-mg acetaminophen tablets were offered four times daily until discharge. The patient-controlled analgesia device was removed when morphine had not been required for at least 6 hours. Donors were discharged when a normal diet was tolerated, and mobilization was adequate.

### Variables

#### Health-related quality of life

HRQoL was our primary outcome and measured using the Short Form-36 Health Survey (SF-36) and the Research and Development 36 (RAND-36), which are well-established tools for assessing QOL in kidney donors [25, 26]. Until 2021, SF-36 questionnaires were administered; subsequently, the SF-36 was replaced by the RAND-36. This change was prompted by the digitalization of the study questions, and it was decided to implement the RAND-36, which is more comprehensive than the SF-36 while maintaining a similar scoring methodology [26]. Questionnaires were distributed preoperatively and at 4, 6, 8, 13, 26, and 52 weeks post-nephrectomy.

The SF-36 and RAND-36 questionnaires comprise 36 items grouped into eight domains: Physical Functioning (PF), Role Physical (RP), Bodily Pain (BP), General Health (GH), Vitality (VT), Social Functioning (SF), Role Emotional (RE), and Mental Health (MH). Each item is scored on a scale, typically from 0 to 100, with higher scores indicating better function and less disability. The domain scores are aggregated to provide a Physical Component Summary (PCS) and a Mental Component Summary (MCS). These summaries are derived by multiplying each domain score by a standard scoring coefficient obtained from factor analysis, reflecting either a physical or mental component. The correlated PCS and MCS were constructed using specific coefficients from the obliquely rotated two-factor solution [27–29]. *Z*-scores were derived using the mean and standard deviation from a representative sample of the general population [30], with factor score coefficients adjusted for the Dutch general population [31]. A higher score reflects a higher HRQoL.

#### Health status

The EQ-5D-3L assessed health levels in five domains: mobility, selfcare, daily activities, pain or discomfort, and anxiety or depression [32]. Donors could choose from three health states per domain: no hindrance, some hindrance, and severe hindrance. The health state corresponds to a standardized index value, which can be calculated for the Dutch general population [33]. Additionally, donors indicated their general health status on a visual analogue scale from 0 to 100, with higher scores indicating better health status. Questionnaires were sent preoperatively and at 2, 6, 13, 26, and 52 weeks post-nephrectomy.

#### Fatigue

Fatigue was measured using the Multidimensional Fatigue Inventory (MFI-20), which has been validated for healthy subjects in the Dutch population [34]. The questionnaire consisted of 20 questions divided over the following five dimensions: general fatigue, physical fatigue, reduced activity, reduced motivation, and mental fatigue. Each dimension is represented by four statements, scored from 1 to 5, resulting in a dimension score ranging from 4 (no fatigue) to 20 (exhausted). Questionnaires were distributed preoperatively and at 2, 6, 13, 26, and 52 weeks post-nephrectomy.

#### Pain

Pain levels were assessed using a visual analogue scale from 0 to 10, where 10 represented ‘the worst pain imaginable’ and 0 represented ‘no pain’ [35]. Pain was measured pre-donation and on days 1, 2, 3, 7, and 14 postoperatively.

### Statistical analysis

We compared mean, standard deviation, and 95% confidence intervals with pre-donation values and between KEP and non-KEP groups. Patients with missing data and those who did not report a pre-donation questionnaire were excluded from the analysis. We tested for differences for each time point between the two groups (KEP and non-KEP), including the entire follow-up period for the between-group analysis. For the within-group analysis, we tested for significant differences between preoperative levels and each follow-up time point. If the data followed a normal distribution based on the Shapiro–Wilk test results [36], we utilized the unpaired *t*-test to compare the means of two independent groups. When normality could not be assumed, we employed the Mann–Whitney *U* test [37, 38] to compare differences between the two independent groups.

Additionally, we employed a mixed-effects regression model for our primary outcome variables to assess differences between KEP and non-KEP over time, accounting for repeated measures within subjects. The dependent variables, PCS and MCS, were log-transformed to meet model assumptions. We tested the models with additional covariates such as age, gender, and type of operation, but did not find any significant influence from these variables. Therefore, the final models included only the time after donation, whether the donor participated in the KEP, and their interaction terms. Model diagnostics, including quantile–quantile (QQ) plots and Cook's distance, were used to evaluate model fit and identify influential observations.

If a significant pre-donation difference exists between groups, subsequent results may be biased towards the group with higher initial scores [39]. Therefore, we used a propensity score matching design to account for significant differences in the primary outcome measure, if present pre-donation. This was the analysis of choice, given the non-randomized observational design of the study, and because we could not assume equal base populations. We utilized a greedy matching algorithm [40] to create a sample population comparable for all baseline characteristics. This approach resulted in matched pairs defined by a standard mean difference of <0.2 for the pre-donation variables.

To determine the sample size needed to detect a meaningful difference in MCS and PCS scores between groups, we referenced the foundational study by Ware *et al*. [41], which provides foundational benchmarks for these measures. In this study, a sample size calculation was performed to assess the required number of participants to detect a 2-point difference within one group over time. The calculation was based on an assumed intertemporal correlation of 0.7, with a power of 80% and a significance level (*α*) of 0.05. Using these parameters, Ware *et al*. determined that a sample of 140 participants would be sufficient to detect a 2-point difference within a single group. However, for comparisons between the KEP donors and the non-KEP donors, our sample size was adequate to detect a clinically significant difference of 5 points in MCS and PCS scores between groups.

For statistical analyses and creation of figures or plots, we used the R software package (R version 4.0.0) [42]. To translate EQ-5D-3L health index values to an index value, we used the STATA software package version 17 [43] using standardized index values for the Dutch general population [44, 43].

## RESULTS

Between 2014 and 2023, 755 living kidney donors underwent a donor nephrectomy at the Erasmus MC Transplant Institute and filled in the questionnaires, with a response rate of 88%. Out of the initial 755 living kidney donors who underwent donor nephrectomy and completed the questionnaires at the Erasmus MC Transplant Institute, 31 donors were excluded from the study because it was not possible to determine their status regarding inclusion in the KEP or non-KEP groups due to missing group assignment data. Finally, a total of 724 donors were included in this study. Donor characteristics are shown in Table 1.

**Table 1:** Donor baseline characteristics.

	KEP	Non-KEP	*P*-value
Baseline			
*n*	121	603	
Gender male (%)	51 (42.1)	274 (45.4)	0.57
Age [mean (SD)]	52.72 (14.29)	52.04 (13.49)	0.62
Donor type (%)			<0.001
Altruistic donor	22 (18.2)	46 (7.6)	
KEP donor	99 (81.8)	0	
Living related	0	312 (51.7)	
Living unrelated	0	245 (40.6)	
Type of donor nephrectomy (%)			0.35
HARP	25 (20.7)	142 (24.1)	
LDN	95 (78.5)	428 (72.5)	
ODN	0	2 (0.3)	
RADN	1 (0.8)	18 (3.1)	

HARP, hand-assisted retroperitoneoscopic donor nephrectomy; LDN, laparoscopic donor nephrectomy; ODN, open donor nephrectomy; RADN, robot-assisted donor nephrectomy.

### Health-related quality of life

**Figure 1:**
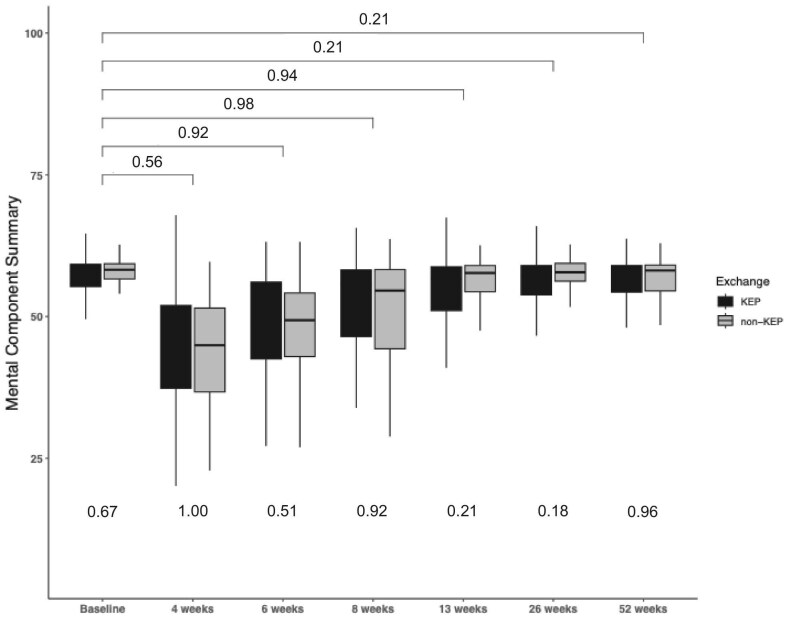
Evolution of the Mental Component Summary over different time periods. Values at the top of the graph represent *P*-values of between significance, which compares the mean of the LDKT Mental Component Summary with pre-donation. The *P*-values at the bottom of the graph represent within significance between KEP and non-KEP.

**Figure 2:**
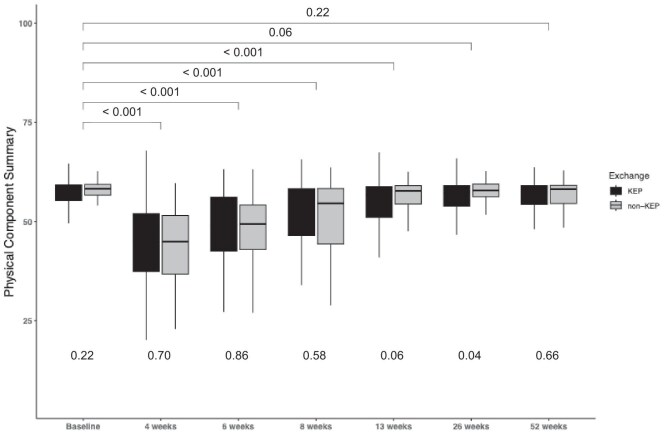
Evolution of the Physical Component Summary over different time periods. Values at the top of the graph represent *P*-values of between significance, which compares the mean of the LDKT Mental Component Summary with pre-donation. The *P*-values at the bottom of the graph represent within significance between KEP and non-KEP.

The HRQoL outcomes are shown in Fig. 1 (MCS) and Fig. 2 (PCS). A detailed statistical overview for HRQoL is shown in Supplementary Data Table S1.

### Mental component summary

The MCS scores were evaluated over 1 year following donor nephrectomy (Fig. 1). The data indicate no significant changes in MCS at any time point compared with pre-donation for both KEP and non-KEP donors. The mixed effects model showed a significant lower MCS at 6- and 8-weeks post-donation (Table 2). Comparative analysis between KEP donors and non-KEP donors revealed no significant differences in MCS at any time point. Using the mixed-effects model for MCS, significant interaction effects were found at 6 months (*P* < 0.05) and 12 months (*P* < 0.01). Specifically, the KEP group had significantly higher MCS scores at these time points compared with the non-KEP group, with percentage changes of 8.7% at 6 months and 11.4% at 12 months (Table 3). No significant pre-donation differences or were observed.

**Table 2:** Comparison of each time point with baseline for PCS and MCS.

Time point	PCS estimate (time versus baseline)	PCS standard error	PCS *P*-value	MCS estimate (time versus baseline)	MCS standard error	MCS *P*-value
4 weeks	−0.22	0.01	<0.01**	−0.02	0.02	0.33
6 weeks	−0.12	0.01	<0.01**	−0.03	0.02	0.04*
8 weeks	−0.06	0.01	<0.01**	−0.05	0.02	0.01*
3 months	−0.01	0.01	0.32	−0.01	0.02	0.55
6 months	0.01	0.01	0.37	−0.00	0.02	0.87
12 months	0.02	0.01	0.18	0.01	0.02	0.74

*Significant at *P* < 0.05; **significant at *P* < 0.01.

Random effects showed a variance of 23.87 (SD 4.89) of the intercept and residual variance of 34.74 (SD 5.89) for the PCS. For the MCS, we calculated a intercept variance of 58.23 (SD 7.63) and a residual variance of 45.15 (SD 6.72).

**Table 3:** Differences between KEP and non-KEP groups at each time point for PCS and MCS in a mixed-effect regression model.

Time point	PCS estimate (KEP–non-KEP)	PCS standard error	PCS *P*-value	MCS estimate (KEP non-KEP)	MCS standard error	MCS *P*-value
Baseline	0.04	0.02	0.06	0.03	0.04	0.48
4 weeks	−0.05	0.03	0.09	−0.06	0.05	0.20
6 weeks	−0.06	0.03	0.04*	−0.03	0.04	0.50
8 weeks	−0.07	0.03	0.03*	0.01	0.05	0.76
3 months	−0.03	0.03	0.32	−0.02	0.04	0.60
6 months	−0.03	0.03	0.35	−0.09	0.04	0.04*
12 months	−0.06	0.03	0.02*	−0.12	0.04	0.01**

*Significant at *P* < 0.05; **significant at *P* < 0.01.

### Physical Component Summary

The PCS scores were significantly lower at 4 weeks, 6 weeks and 2 months compared with pre-donation for both KEP and non-KEP donors (Fig. 2), with all time points showing significant decreases in PCS scores (*P* < 0.001). After we applied the mixed-effects model, the PCS was also significantly decreased up to 8 weeks post-donation (Table 2). Between-group comparisons showed no significant differences in PCS scores between KEP and non-KEP donors at other time points. Using the mixed-effects regression model for PCS, significant differences were observed between the KEP and non-KEP groups at 6 and 8 weeks (all *P* < 0.05). The analysis showed that the non-KEP group had significantly lower PCS scores at these time points compared with the KEP group, with percentage changes of 19.8% at 4 weeks, 11.4% at 6 weeks, and 5.9% at 8 weeks. Additionally, a significant interaction effect was found at 12 months (*P* < 0.05), with the PCS score in the non-KEP group being 5.9% lower than in the KEP group. No significant pre-donation differences were observed (Table 3).

Because surgical practice changed over time, we performed a sensitivity analysis to see whether this change in practice resulted in a change in HRQoL. Supplementary Data Fig. S1 shows that the PCS did not differ between hand-assisted retroperitoneoscopic donor nephrectomy (HARP) and laparoscopic donor nephrectomy (LDN), while the MCS was significantly higher in HARP pre-donation and at 6, 13, and 26 weeks. After propensity score matching (Supplementary Data Fig. S2) there was no significant difference between HARP and LDN.

### Health status

**Figure 3:**
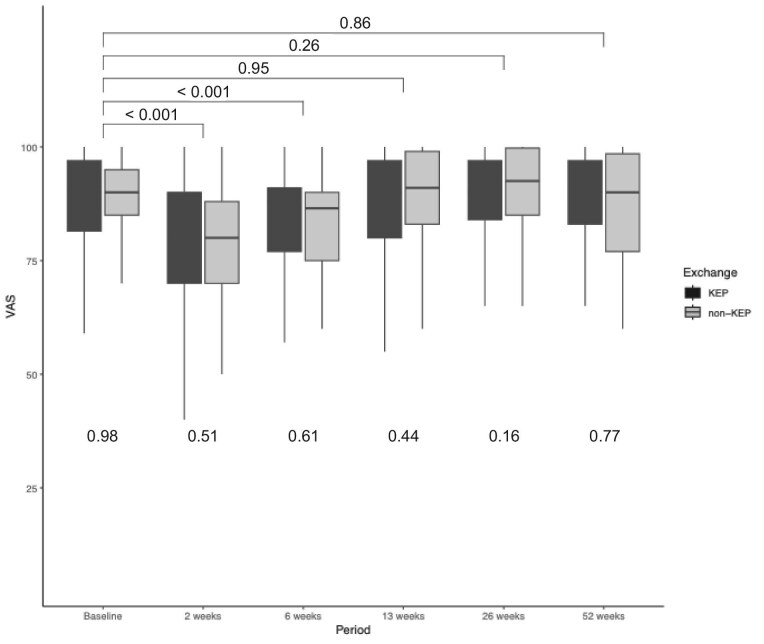
Self-rated visual analogue (VAS) score of the EQ-5D-3L. Values at the top of the graph represent *P*-values of between significance, which compares the mean of the LDKT self-rated quality of life with pre-donation. The *P*-values at the bottom of the graph represent within significance between KEP and non-KEP.

The scores of the EQ-5D-3L to measure health status are shown in Supplementary Data Table S2 and the self-rated QoL of the donors is shown in Fig. 3.

The index values were significantly lower than pre-donation 2 weeks postoperatively, with a *P*-value of <0.001. We found a significant difference on the EQ-5D-3L between KEP and non-KEP at 6 weeks and 12 months in favour of KEP. The results of the visual analogue scale are shown in Fig. 3. Donors experienced significantly decreased self-rated health at 2 and 6 weeks postoperatively compared with pre-donation. We did not find a difference in the means of self-rated health between KEP and non-KEP.

### Fatigue

**Figure 4:**
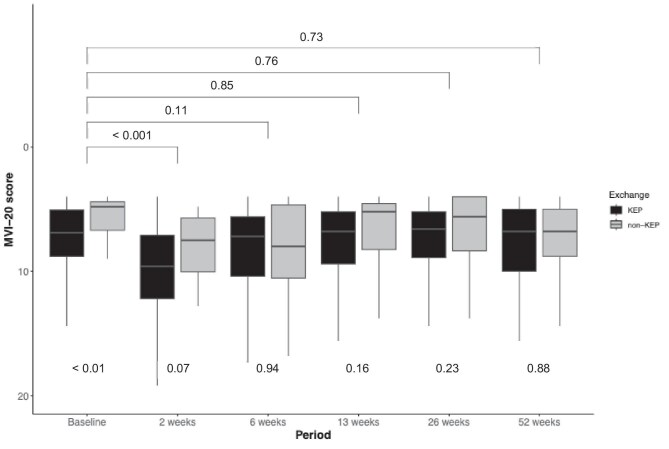
Average MVI-20 score stratified by donation type measuring fatigue in the cohort per time point. Values at the top of the graph represent *P*-values of between significance, which compares the mean of the LDKT average MVI-20 score with pre-donation. The *P*-values at the bottom of the graph represent within significance between KEP and non-KEP.

The MVI-20 scores are shown in Fig. 4, and an extensive description of the mean scores per domain with statistical details is shown in Supplementary Data Table S3. Both KEP and non-KEP groups showed significantly higher MVI-20 scores at 2 weeks postoperatively compared with pre-donation. However, the scores at 6, 13, 26, and 52 weeks postoperatively were not significantly different from pre-donation for both groups.

The within-group analysis showed no significant post-donation differences between KEP and non-KEP; however, pre-donation the non-KEP group scored significantly better. Additionally, post-donation there were no significant differences found between KEP and non-KEP on the five separate domains of fatigue.

### Pain

**Figure 5:**
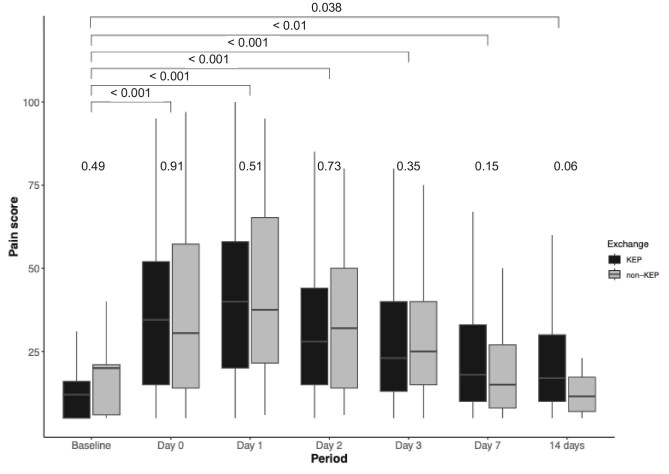
Average self-rated pain level on a visual analogue scale. Values at the top of the graph represent *P*-values of between significance, which compares the mean of the LDKT self-rated pain level with pre-donation. The *P*-values beneath the previous ones represent within significance between KEP and non-KEP.

Figure 5 and Supplementary Data Table S4 show that up to 14 days post-operatively the pain scores were significantly higher compared with pre-nephrectomy. There were no significant differences between KEP and non-KEP groups at any postoperative time point.

## DISCUSSION

The results of our study provide important insights into the HRQoL of living kidney donors participating in KEPs. Our findings suggest that KEP participation does not adversely affect HRQoL compared with direct donation to an intended recipient. In fact, at certain time points donors in KEPs reported better HRQoL outcomes, as evidenced by higher scores on both physical and mental health measures. These results are particularly reassuring for potential donors who might be hesitant to participate in KEPs due to concerns about their physical or emotional well-being.

By demonstrating that donors in KEPs generally experience similar or even better HRQoL outcomes, our study provides strong support for the expansion of KEPs as an effective strategy to increase living donor kidney transplantation rates. The observed temporary declines in some HRQoL domains, such as fatigue and self-reported health, quickly returned to pre-donation levels, underscoring the resilience of donors and the manageable nature of post-donation recovery, regardless of the donation type. This information could help alleviate concerns among potential donors about the impact of participating in a KEP, which may contribute to higher participation rates and ultimately expand the donor pool.

Furthermore, our study adds novel value to the existing literature by specifically comparing HRQoL outcomes between KEP and non-KEP donors, a comparison that has been underexplored. Previous studies already showed that the HRQoL of living kidney donors is generally good, just slightly lower compared with pre-donation levels but comparable to the general population [16]. Fatigue was found to be a major complaint, which existed for a long period after donor nephrectomy [16, 45, 46]. However, in our study we found that living kidney donors returned to pre-donation fatigue levels after just 2 weeks without a difference between KEP and non-KEP.

Our findings suggest that donors who participate in KEPs do not experience a significant decline in HRQoL compared with those who donated directly to their intended recipient. These results are reassuring for living kidney donors, which could encourage more individuals to consider donating a kidney through this approach. Our study has novel value, given that prior to the start of this study we performed a systematic review and meta-analysis [47] and found no studies on living kidney donor HRQoL that distinguished between KEP and non-KEP donation, suggesting that this study makes a valuable contribution to this area of research. Some papers compared KEP with non-KEP in terms of perception of coercion [48] and the amount of psychosocial support [49], and both studies found no differences between the two groups. Bourkas and Achille [50] showed that the donors participating in the KEP live with co-inflated responsibility, guilt, worries, and indebtedness with gratitude toward known and anonymous donors.

In this study cohort, as per the national protocol, donors travelled to the recipient centre, which means that the donor nephrectomy and kidney transplantation took place in the same centre. However, other options exist when KEP is performed between centres. The alternative option is to transport the kidney from the donor to the recipient, in the same way that many deceased donor organs are currently transported [17]. The donor travelling to the recipient's centre in the Netherlands contrasts with practices in the USA and UK, where the kidney is usually transported to the recipient centre after living donor nephrectomy if part of KEP. Travelling to the recipient's centre means that the donor is not in proximity of the intended (emotionally and/or genetically related) recipient and is treated by an unfamiliar surgical team, which could lead to additional adverse effects on the HRQoL. However, in this study we demonstrated that there is no negative impact of KEP participation on HRQoL. Our findings from the mixed-effects models suggest that KEP donors may have better HRQoL outcomes at certain time points compared with non-KEP donors. Our results are likely to be generalizable even for countries with KEPs where the kidney is transported; the HRQoL will likely be comparable or even higher compared with our results. This is because the donation process may be perceived as more straightforward for the donor in these countries, potentially reducing psychological stress caused by logistics (such as the donor travelling) or emotional burden (due to proximity to the intended recipient). Future research is needed to confirm this hypothesis. Based on our data, we encourage countries with an LDKT programme to increase the number of LDKTs by implementing a KEP, if not yet in place. When comparing the different options when KEP is performed between centres, the donor's HRQoL is not a reason to opt against the donor travelling to the recipient's centre for the live donor nephrectomy, based on the results from our study. As shown in previous studies [11, 42], transporting the kidney does not result in impaired graft outcomes after LDKT. Therefore, transporting the kidney after living donor nephrectomy and travelling of the donor to the recipient's centre are both good options for KEPs.

One limitation of our study is that we used data from a single centre. In this study we did not evaluate other potential factors that could impact the HRQoL of donors, such as socio-economic status, racial background, or employment status, due to the unavailability of these data. In the Netherlands, privacy regulations strictly limit the collection of certain personal information, including socio-economic and racial data, for research purposes to protect individual privacy. Consequently, we were unable to include these variables in our analysis.

While acknowledging this as a limitation, we believe our findings remain robust, given that the primary focus of this study was on the HRQoL outcomes directly associated with the donation process itself. Future research that can access broader datasets or utilize different methodologies to collect socio-economic and demographic data might provide further insights into the potential influence of these factors on HRQoL in living kidney donors. Furthermore, missing data may have led to sampling bias in our study. Finally, since the Netherlands is a relatively small and densely populated country, these results may differ in large countries where donors must travel substantially further. These factors could potentially confound the results and should be considered in future studies.

Our findings are relevant for several reasons. Firstly, they provide reassurance to potential living kidney donors who may be hesitant to participate in KEPs due to concerns about the impact on their psychological or emotional factors. Our findings are in line with research at commencement of the KEP in our centre showing that no additional psychosocial support for KEP participants is needed [51]. These findings take away another potential barrier to the expansion of KEP and strengthen the argument for KEP as an effective strategy to increase living donor kidney transplantation rates.

Finally, while the risks associated with living kidney donation are low [14], it is important to monitor donors for any adverse effects that may arise following the procedure. Further studies should investigate the long-term impact of KEPs on the donor's HRQoL after 1 year post-donation in a multicentre study and study risk factors for impaired HRQoL in living kidney donors participating in KEP.

## CONCLUSION

We here provide evidence that the decline in the living kidney donor's HRQoL following nephrectomy is temporary and restores to pre-donation levels after 3 months for both KEP and non-KEP LDKT.

Our study’s most significant contribution is demonstrating that living kidney donors who participate in KEP do not experience lower HRQoL, even when they need to travel to another hospital and donate their kidney to a recipient other than their original intended. This finding takes away another potential barrier to the expansion of KEP and strengthens the argument for KEP as an effective strategy to increase LDKT rates.

## Supplementary Material

sfae374_Supplemental_File

## Data Availability

The data underlying this article are available in the article and in its online supplementary material. The data underlying this article will be shared on reasonable request to the corresponding author.
