# Supplementary material for: Health-related quality of life in living kidney donors participating in kidney exchange programmes
Source: Clin Kidney J. 2024 Nov 23;18(2):sfae374. doi: 10.1093/ckj/sfae374 (PMC11799774; doi:10.1093/ckj/sfae374)
Supplement: sfae374_Supplemental_File [file sfae374_supplemental_file.docx]

| **Time after nephrectomy** | **Group** | **Mean MCS** | **95% CI MCS** | **Within p-value MCS** | **Between p-value MCS** | **Mean PCS** | **95% CI PCS** | **Within p-value PCS** | **Between p-value PCS** |
| --- | --- | --- | --- | --- | --- | --- | --- | --- | --- |
| Pre-donation | KEP | 51.90 | 50.70 - 53.00 | - | 0.67 | 52.90 | 52.00 - 53.70 | - | 0.22 |
|  | non KEP | 52.80 | 52.30 - 53.20 |  |  | 52.50 | 52.20 - 52.90 |  |  |
| 4 weeks | KEP | 49.70 | 46.60 - 52.90 | 0.56 | 1.00 | 43.50 | 41.30 - 45.70 | < 0.001 | 0.70 |
|  | non KEP | 50.40 | 49.20 - 51.60 |  |  | 43.80 | 42.70 - 44.80 |  |  |
| 6 weeks | KEP | 51.70 | 49.30 - 54.20 | 0.92 | 0.51 | 48.60 | 46.50 - 50.80 | < 0.001 | 0.86 |
|  | non KEP | 50.30 | 49.20 - 51.40 |  |  | 48.90 | 48.10 - 49.80 |  |  |
| 8 weeks | KEP | 52.50 | 49.60 - 55.50 | 0.98 | 0.92 | 50.90 | 48.10 - 53.70 | < 0.001 | 0.58 |
|  | non KEP | 51.40 | 49.90 - 53.00 |  |  | 52.20 | 51.10 - 53.20 |  |  |
| 3 months | KEP | 52.30 | 49.80 - 54.80 | 0.98 | 0.21 | 55.90 | 54.40 - 57.30 | < 0.001 | 0.06 |
|  | non KEP | 51.10 | 50.10 - 52.10 |  |  | 54.10 | 53.40 - 54.90 |  |  |
| 6 months | KEP | 51.40 | 48.10 - 54.70 | 0.21 | 0.18 | 57.60 | 56.80 - 58.50 | 0.06 | 0.04 |
|  | non KEP | 51.70 | 50.80 - 52.70 |  |  | 55.50 | 54.80 - 56.20 |  |  |
| 12 months | KEP | 50.40 | 47.20 - 53.70 | 0.21 | 0.96 | 55.40 | 53.30 - 57.50 | 0.22 | 0.66 |
|  | non KEP | 52.40 | 51.50 - 53.40 |  |  | 55.70 | 55.10 - 56.40 |  |  |

Table 1. MCS and PCS scores of living kidney donors pre- and postoperative.

The within values represent the level of significance between pre- and post-nephrectomy of the SF/RAND-36 values. The between values represent the level of significance within a time-point between KEP and non KEP. KEP = kidney exchange programme, MCS = mental component summary, PCS = physical component summary, SD = standard deviation.

Figure 1. Evolution of respectively the physical and mental component summary over different time periods.
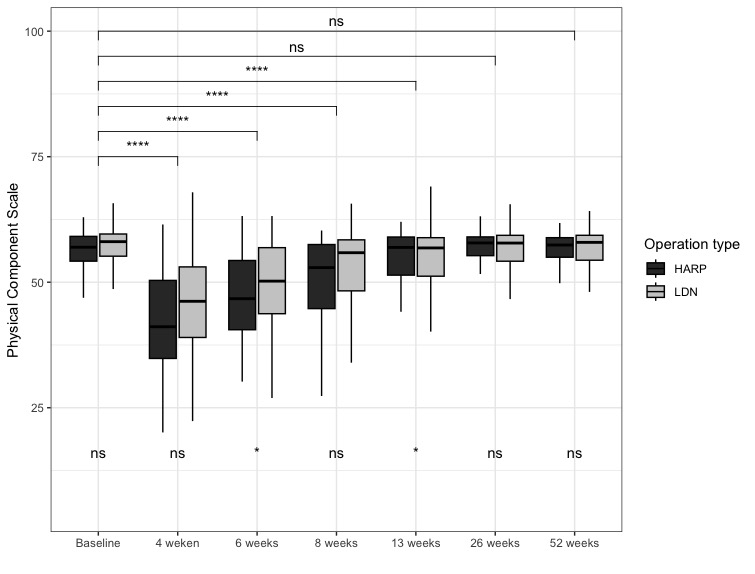

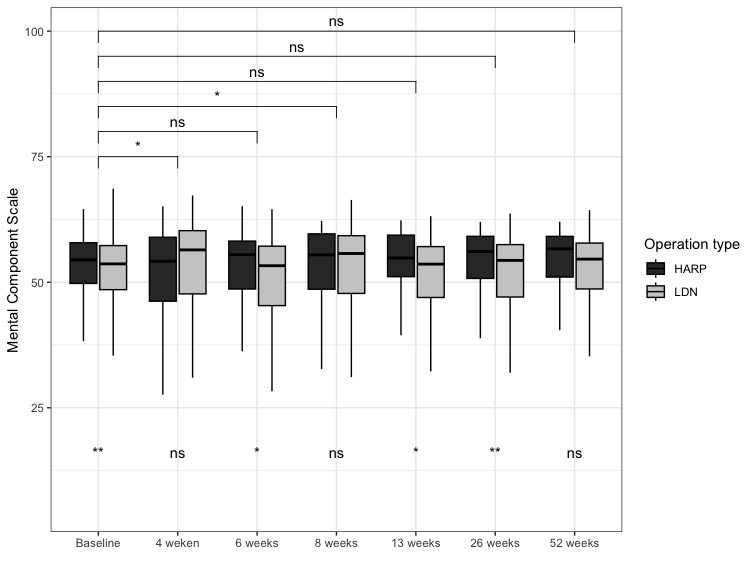


Values at the top of the graph represents p-values of between significance which compares the mean of the LDKT mental component summary scores to the pre-donation. The symbols at the bottom of the graph represent within significance between HARP and LDN. ns: p > 0.05, *: p < 0.05, **: p < 0.01, ***: p < 0.001, ****: p < 0.0001

Figure 2. Evolution of respectively the physical and mental component summary over different time periods after propensity scored matching.
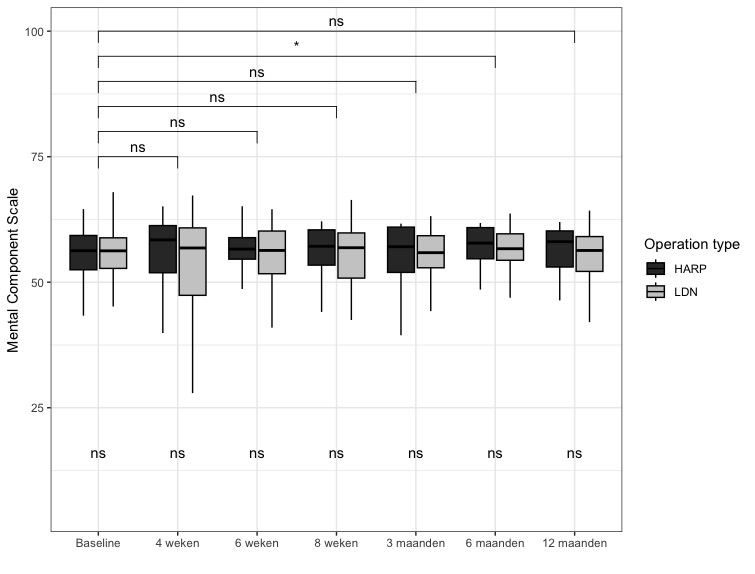

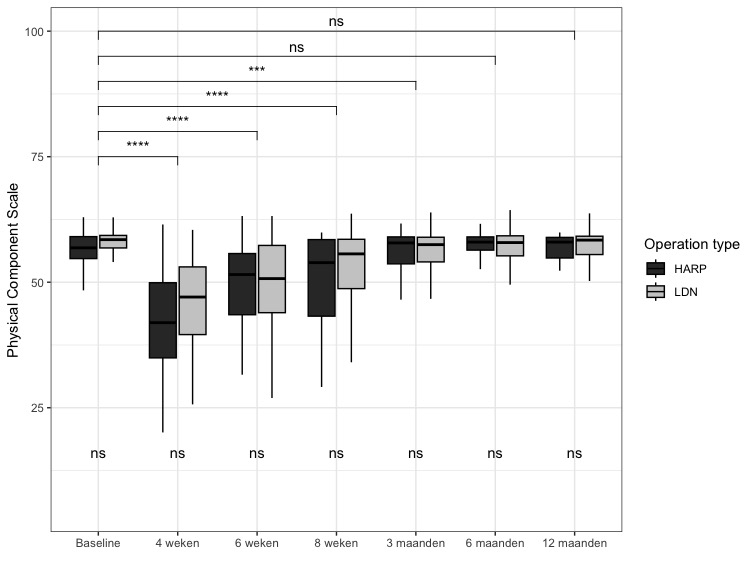


Values at the top of the graph represents p-values of between significance which compares the mean of the LDKT mental component summary scores to the pre-donation. The symbols at the bottom of the graph represent within significance between HARP and LDN. ns: p > 0.05, *: p < 0.05, **: p < 0.01, ***: p < 0.001, ****: p < 0.0001

Table 2. The standardized index values of the mean EQ-5D-3L health states per donation type.

| **Time** | **Group** | **Mean** | **SD** | **95% CI** | **Between p-value** | **Between p-value** | **Mean Difference** |
| --- | --- | --- | --- | --- | --- | --- | --- |
| Pre-donation | KEP | 0.89 | 0.10 | 0.88 - 0.90 | 0.15 | NA | NA |
|  | non-KEP | 0.91 | 0.08 | 0.89 - 0.93 |  |  |  |
| 14 days | KEP | 0.75 | 0.15 | 0.73 - 0.77 | 0.72 | < 0.001 | -0.14 |
|  | non-KEP | 0.76 | 0.14 | 0.72 - 0.80 |  |  | -0.15 |
| 6 weeks | KEP | 0.87 | 0.11 | 0.85 - 0.88 | 0.03 | 0.32 | -0.03 |
|  | non-KEP | 0.78 | 0.17 | 0.70 - 0.87 |  |  | -0.13 |
| 3 months | KEP | 0.89 | 0.11 | 0.88 - 0.90 | 0.81 | 0.55 | -0.00 |
|  | non-KEP | 0.90 | 0.10 | 0.87 - 0.92 |  |  | -0.01 |
| 6 months | KEP | 0.91 | 0.09 | 0.90 - 0.92 | 0.84 | 0.10 | 0.02 |
|  | non-KEP | 0.91 | 0.08 | 0.89 - 0.93 |  |  | 0.00 |
| 12 months | KEP | 0.91 | 0.09 | 0.90 - 0.92 | 0.03 | 0.27 | 0.02 |
|  | non-KEP | 0.86 | 0.19 | 0.81 - 0.90 |  |  | -0.06 |

KEP: kidney exchange programme, MD: mean difference, VAS: the EQ VAS records the patient’s self-rated health on a vertical visual analogue scale, where the endpoints are labelled ‘The best health you can imagine’ and ‘The worst health you can imagine’, with a higher score corresponding to better health.

Table 3. The values of the MVI-20 per domain.

| **Time after nephrectomy** | **Group** | **Domain** | **Mean** | **SD** | **95% CI** | **Within p-value** | **Between p-value** |
| --- | --- | --- | --- | --- | --- | --- | --- |
| Pre-donation | Non KEP | General | 7.54 | 3.95 | 6.87 - 8.21 | NA |  |
|  |  | Physical | 7.13 | 4.35 | 6.4 - 7.87 |  |  |
|  |  | Activity | 8.37 | 4.44 | 7.62 - 9.13 |  |  |
|  |  | Motivation | 8.05 | 3.76 | 7.41 - 8.68 |  |  |
|  |  | Cognitive | 8.16 | 4.03 | 7.48 - 8.84 |  |  |
|  | KEP | General | 5.13 | 1.31 | 4.49 - 5.76 |  | 0.02 |
|  |  | Physical | 5.20 | 1.66 | 4.4 - 6 |  | 0.17 |
|  |  | Activity | 6.00 | 2.99 | 4.56 - 7.44 |  | 0.02 |
|  |  | Motivation | 6.00 | 2.13 | 4.97 - 7.03 |  | 0.04 |
|  |  | Cognitive | 6.19 | 2.54 | 4.97 - 7.41 |  | 0.07 |
| 2 weeks | Non-KEP | General | 10.37 | 4.14 | 9.64 - 11.1 | < 0.001 |  |
|  |  | Physical | 10.40 | 4.26 | 9.65 - 11.15 |  |  |
|  |  | Activity | 11.43 | 4.18 | 10.69 - 12.16 |  |  |
|  |  | Motivation | 8.90 | 3.88 | 8.22 - 9.58 |  |  |
|  |  | Cognitive | 8.28 | 3.99 | 7.57 - 8.98 |  |  |
|  | KEP | General | 8.44 | 3.18 | 6.8 - 10.07 |  | 0.08 |
|  |  | Physical | 8.38 | 3.44 | 6.61 - 10.14 |  | 0.07 |
|  |  | Activity | 9.81 | 3.67 | 7.92 - 11.7 |  | 0.15 |
|  |  | Motivation | 7.88 | 3.07 | 6.29 - 9.46 |  | 0.40 |
|  |  | Cognitive | 6.25 | 2.74 | 4.84 - 7.66 |  | 0.05 |
| 6 weeks | Non KEP | General | 8.97 | 4.29 | 8.23 - 9.71 | 0.11 |  |
|  |  | Physical | 8.27 | 4.10 | 7.56 - 8.97 |  |  |
|  |  | Activity | 9.33 | 9.57 | 7.68 - 10.98 |  |  |
|  |  | Motivation | 7.44 | 3.61 | 6.82 - 8.06 |  |  |
|  |  | Cognitive | 7.71 | 3.61 | 7.09 - 8.33 |  |  |
|  | KEP | General | 9.50 | 5.56 | 6.73 - 12.27 |  | 0.99 |
|  |  | Physical | 8.38 | 4.16 | 6.31 - 10.44 |  | 0.99 |
|  |  | Activity | 9.06 | 5.04 | 6.56 - 11.57 |  | 0.99 |
|  |  | Motivation | 7.50 | 3.50 | 5.76 - 9.24 |  | 0.93 |
|  |  | Cognitive | 7.13 | 4.18 | 5.05 - 9.20 |  | 0.38 |
| 3 months | Non KEP | General | 8.51 | 4.13 | 7.82 - 9.21 | 0.85 |  |
|  |  | Physical | 7.36 | 3.69 | 6.74 - 7.99 |  |  |
|  |  | Activity | 7.34 | 3.53 | 6.74 - 7.93 |  |  |
|  |  | Motivation | 6.95 | 3.04 | 6.43 - 7.46 |  |  |
|  |  | Cognitive | 7.44 | 3.87 | 6.79 - 8.10 |  |  |
|  | KEP | General | 7.31 | 4.32 | 5.29 - 9.33 |  | 0.18 |
|  |  | Physical | 6.00 | 2.73 | 4.72 - 7.28 |  | 0.13 |
|  |  | Activity | 6.94 | 3.15 | 5.46 - 8.41 |  | 0.66 |
|  |  | Motivation | 6.25 | 2.74 | 4.97 - 7.53 |  | 0.32 |
|  |  | Cognitive | 6.75 | 4.37 | 4.70 - 8.80 |  | 0.26 |
| 6 months | Non KEP | General | 8.37 | 3.91 | 7.75 - 8.99 | 0.68 |  |
|  |  | Physical | 7.89 | 9.95 | 6.31 - 9.46 |  |  |
|  |  | Activity | 7.98 | 9.54 | 6.47 - 9.49 |  |  |
|  |  | Motivation | 6.93 | 2.87 | 6.48 - 7.39 |  |  |
|  |  | Cognitive | 9.74 | 15.68 | 7.26 - 12.22 |  |  |
|  | KEP | General | 12.48 | 22.18 | 3.11 - 21.84 |  | 0.37 |
|  |  | Physical | 6.38 | 3.01 | 5.11 - 7.65 |  | 0.20 |
|  |  | Activity | 7.57 | 3.92 | 5.92 - 9.23 |  | 0.96 |
|  |  | Motivation | 6.48 | 3.17 | 5.14 - 7.82 |  | 0.29 |
|  |  | Cognitive | 6.52 | 3.36 | 5.11 - 7.94 |  | 0.24 |
| 12 months | Non KEP | General | 8.61 | 3.94 | 7.98 - 9.23 | 0.73 |  |
|  |  | Physical | 7.24 | 2.97 | 6.77 - 7.71 |  |  |
|  |  | Activity | 8.57 | 10.30 | 6.93 - 10.20 |  |  |
|  |  | Motivation | 7.12 | 2.88 | 6.66 - 7.58 |  |  |
|  |  | Cognitive | 7.19 | 3.53 | 6.63 - 7.75 |  |  |
|  | KEP | General | 14.61 | 24.35 | 4.33 - 24.89 |  | 0.80 |
|  |  | Physical | 8.28 | 4.32 | 6.45 - 10.10 |  | 0.54 |
|  |  | Activity | 8.39 | 4.35 | 6.55 - 10.23 |  | 0.72 |
|  |  | Motivation | 7.39 | 3.62 | 5.86 - 8.92 |  | 0.98 |
|  |  | Cognitive | 6.50 | 3.96 | 4.83 - 8.17 |  | 0.28 |

Within values represent the level of significance between post-donation time-points and pre-donation. Between values show the level of significance between KEP and non-KEP. KEP: kidney exchange programme. KEP: kidney exchange programme, SD: standard deviation.

| **Time after nephrectomy** | **Group** | **Mean** | **SD** | **95% CI Interval** | **Between P-values** | **Mean Difference Compared to Pre-donation** | **Within P-values** |
| --- | --- | --- | --- | --- | --- | --- | --- |
| Pre-operatively | KEP | 1.80 | 7.27 | 1.08 - 2.52 | 0.18 | - | - |
|  | non KEP | 1.34 | 5.69 | 0.09 - 2.59 |  |  |  |
| Day 0 | KEP | 20.13 | 24.63 | 17.6 - 22.66 | 0.34 | 18.33 | < 0.001 |
|  | non KEP | 23.70 | 26.67 | 17.44 - 29.97 |  | 22.36 |  |
| Day 1 | KEP | 30.50 | 27.06 | 27.73 - 33.27 | 0.58 | 28.70 | < 0.001 |
|  | non KEP | 33.27 | 28.74 | 26.62 - 39.93 |  | 31.93 |  |
| Day 2 | KEP | 23.07 | 22.88 | 20.72 - 25.42 | 0.88 | 21.27 | < 0.001 |
|  | non KEP | 23.78 | 24.37 | 18.05 - 29.51 |  | 22.44 |  |
| Day 3 | KEP | 19.44 | 22.06 | 17.15 - 21.73 | 0.61 | 17.64 | < 0.001 |
|  | non KEP | 18.85 | 21.21 | 13.94 - 23.76 |  | 17.51 |  |
| Day 7 | KEP | 12.41 | 17.15 | 10.61 - 14.21 | 0.59 | 10.61 | < 0.01 |
|  | non KEP | 10.90 | 15.80 | 7.24 - 14.56 |  | 9.56 |  |
| Day 14 | KEP | 10.76 | 15.83 | 8.65 - 12.87 | 0.36 | 8.96 | 0.04 |
|  | non KEP | 7.87 | 12.49 | 4.39 - 11.34 |  | 6.53 |  |

Table 4. The self-reported pain levels scored on a visual analog scale.

Within values represent the level of significance between post-donation time-points and pre-donation. Between values show the level of significance between KEP and non-KEP. KEP: kidney exchange programme, SD: standard deviation.
